# Differences between ethnic groups in self‐reported use of e‐cigarettes and nicotine replacement therapy for cutting down and temporary abstinence: a cross‐sectional population‐level survey in England

**DOI:** 10.1111/add.15431

**Published:** 2021-03-18

**Authors:** Emma Beard, Jamie Brown, Sarah E. Jackson, Harry Tattan‐Birch, Lion Shahab

**Affiliations:** ^1^ Department of Behavioural Science and Health University College London London UK

**Keywords:** Cigarettes, e‐cigarettes, ethnicity, smoking, STS, trends

## Abstract

**Background and aims:**

The National Institute for Health and Care Excellence (NICE) has called for research into tobacco harm reduction across ethnicities, genders and socio‐economic status. Although there is increasing research focused on the latter two, relatively few studies have considered ethnic variations. Therefore this study aimed to assess (i) the association between ethnicity and use of e‐cigarettes and nicotine replacement therapy (NRT) for temporary abstinence and cutting down, and (ii) trends in prevalence of these over time.

**Design:**

Repeated cross‐sectional household survey.

**Setting:**

England.

**Participants:**

Between April 2013 and September 2019, data were collected on 24 114 smokers, 16+ of age, taking part in the Smoking Toolkit Study (STS).

**Measurements:**

Ethnicity coding included: White, mixed/multiple ethnic group, Asian, Black and Arab/other ethnic group. Smokers reported whether they were currently using e‐cigarettes and/or NRT for cutting down or during periods of temporary abstinence.

**Findings:**

Odds of e‐cigarette use for cutting down and temporary abstinence were significantly lower among those of Asian ethnicity (OR = 0.79, 95% CI = 0.66–0.93) and Arab/other ethnicity (OR = 0.58, 95% CI = 0.40–0.83) compared with White ethnicity. Those of mixed/multiple ethnicity had higher odds for NRT us (OR = 1.42, 95% CI = 1.04–1.94) compared with those of White ethnicity. Trend analysis indicated that for White ethnicity, e‐cigarette use by smokers for cutting down and temporary abstinence followed an ‘inverse S’ shaped cubic curve indicating an overall rise, whereas NRT use followed an ‘S’ shaped cubic curve, indicating an overall decline. For mixed/multiple ethnicity a similar trend was found for NRT use only, with other ethnicities showing no statistically significant trends (suggesting relative stability over time).

**Conclusions:**

In England, e‐cigarette use by smokers for cutting down and temporary abstinence is less common among Asian and Arab/other ethnicity smokers compared with White smokers. Smokers of mixed/multiple ethnicity are the most likely to be using NRT compared with other ethnic groups for cutting down and temporary abstinence. E‐cigarette use by smokers for cutting down and temporary abstinence has increased over time among White smokers, whereas prevalence in other ethnic groups has remained stable.

## BACKGROUND

Although E‐cigarettes are deemed to be substantially safer than combustible cigarettes [1, 2, 3, 4, 5, 6], regulation of electronic cigarettes varies across countries ranging from no regulation to complete bans. England has an intermediate position that allows tightly regulated consumer sales. Regulations include a cross‐border advertising ban, age‐of‐sale restrictions, minimum quality and safety standards and a notification system overseen by the Medicines and Healthcare Products Regulatory Agency (MHRA) [7]. England has quite a liberal approach to nicotine replacement therapy (NRT), which is available both over‐the‐counter and as a medication and includes an indication for harm reduction. Although many smokers use these products as a smoking cessation aid, an increasing number are opting for harm reduction purposes [8]. Harm reduction involves any attempt to reduce the harm from smoking without the complete elimination of all tobacco constituents [9]; the most common forms being the use of Alternative Nicotine Delivery Systems (ANDS) for smoking reduction (i.e. cutting down) and temporary abstinence (i.e. periods of time when one is unable to smoke) [10, 11].

NRT has been licensed as an aid for cutting down and temporary abstinence in the United Kingdom (UK) since December 2009, following evidence that use for such purposes increases the propensity of smokers to quit smoking [12, 13, 14]. In contrast, none of the e‐cigarette products currently on the market in the United Kingdom have been licensed as medicines and are instead regulated under the revised European Union (EU) Tobacco Product Directive (2016) [15]. This means that they must adhere to product specification and marketing restrictions. There is mixed literature on the use of e‐cigarettes and NRT for cutting down and temporary abstinence [16, 17]. Although clinical trials show that the use of NRT for smoking reduction can result in significant reductions in cigarette consumption and harm; outside of this carefully controlled setting reductions in cigarette consumption are negligible [4, 9, 11, 14, 18]. In the United Kingdom, at a population level the prevalence of current smokers using e‐cigarette for cutting down and temporary abstinence is positively associated with the prevalence of overall quit rates but not associated with reductions in cigarette consumption [19, 20]. There is also evidence that use of NRT for cutting down and temporary abstinence may increase the likelihood of making a quit attempt and achieving cessation [9, 14]. Evidence is also emerging from clinical trials that the same may be true for e‐cigarette use [21]. As a consequence, use of e‐cigarettes and NRT for cutting down and temporary abstinence offers a potentially useful tool for reducing smoking rates [5, 22].

To help inform policy recommendations, it is important to explore not only the efficacy and effectiveness of ANDS for cutting down and temporary abstinence but also their use by different socio‐demographic groups. The National Institute for Health and Care Excellence (NICE) has called for research into tobacco harm reduction across ethnicities, genders and socio‐economic status [23]. Although there is increasing research focused on the latter two, relatively few studies have considered ethnic variation [24, 25]. Therefore, this study aims to examine ethnic differences and trends in e‐cigarette and NRT use for cutting down and temporary abstinence using data from a nationally representative survey in England, the Smoking Toolkit Study, over a 7‐year period between 2013 and 2019.

Studies on the prevalence of e‐cigarette use across different ethnic groups have shown that ethnic minorities are less likely than those of White ethnicity to use e‐cigarettes to quit smoking or for cutting down and temporary abstinence [26, 27, 28, 29, 30, 31, 32, 33]. In part, this may be explained by higher perceived harms of e‐cigarettes [26, 34]. Sociocultural influences, social norms and targeted marketing strategies may also play a role [35, 36]. It is also conceivable that e‐cigarettes provide a better substitute for cigarettes than for shisha and smokeless tobacco [37].

However, the majority of these studies to date were conducted in the United States and focused mainly on those of Black, Hispanic and White ethnicity. Results cannot be presumed to extrapolate to other countries with different ethnic profiles. In England, ethnic minorities currently represent approximately 14% of the total population, with those of Asian and Black ethnicity comprising ~11% of the population [38].

The aims of this study are: 1) to estimate and test the strength of association between ethnicity and the use of e‐cigarettes and NRT for cutting down and temporary abstinence by current smokers; and 2) to assess trends over time in use of e‐cigarettes and NRT for cutting down and temporary abstinence by current smokers as a function of ethnicity.

## DESIGN

Data came from the Smoking Toolkit Study (STS) [39]. The STS involves monthly cross‐sectional household computer‐assisted interviews, conducted by Ipsos Mori, of ∼1700 adults of 16+ years of age in England. Data were used from April 2013 (the first wave to include questions on both ethnicity and e‐cigarette use) until September 2019 (the most recent data available at the time of analysis). Analyses focused on participants who reported smoking cigarettes (including hand‐rolled) daily or occasionally at the time of the survey (‘current smokers’).

## MEASURES

### Covariates

Data were collected on individuals’ smoking status, age, gender and socio‐economic status. Socio‐economic status was measured using social grade and housing tenure.

Social grade was measured using the British National Readership Survey (NRS) Social Grade Classification Tool [40]: AB (higher managerial, administrative or professional), C1 (supervisory or clerical and junior managerial, administrative or professional), C2 (skilled manual workers), D (semi‐skilled and unskilled manual workers) and E (casual or lowest grade workers, pensioners and others who depend on the welfare state for their income).

Housing tenure was measured in six categories (mortgaged, owned outright, rented from local authority, rented from private landlord, belongs to housing association and other). For analysis, we dichotomised responses to distinguish between owner‐occupied (owned outright or being bought with a mortgage) and other housing tenures [41].

The Heaviness of Smoking Index (HSI) was also assessed as a measure of cigarette dependence [42].

### Independent variable

Participants were asked to report their ethnicity using a question adopted by the 2011 England and Wales census [38], with responses categorized into the following five ethnic groups: White (White British, White Irish, White Gypsy/Traveller, White other); mixed/multiple ethnic group (Mixed White/Black Caribbean, White/Black African, White/Asian, White other); Asian (Asian Indian, Asian Pakistani, Asian Bangladeshi, Asian Chinese, Asian other); Black (Black African, Black Caribbean, Black other); Arab/other ethnic group (Arab, other).

### Dependent variables

Smokers were asked the following questions to assess use of e‐cigarettes and NRT:

1. Which, if any, of the following are you currently using to help you cut down the amount you smoke? Response options: nicotine gum, nicotine replacement lozenges\tablets, nicotine replacement inhaler, nicotine replacement nasal spray, nicotine patch, electronic cigarette, nicotine mouth spray, other.
2. Do you regularly use any of the following in situations when you are not allowed to smoke? Response options: nicotine gum, nicotine replacement lozenges\tablets, nicotine replacement inhaler, nicotine replacement nasal spray, nicotine patch, electronic cigarette, nicotine mouth spray, other.Smokers who reported that they used one of the NRT products in relation to question 1 or 2 were classed as NRT users and those who reported that they used e‐cigarettes were classed as e‐cigarette users. Dual users were included as both e‐cigarette users and NRT users.

## ANALYSIS

The analysis plan was pre‐registered on the Open Science Framework (https://osf.io/72xsf/). An amendment was made to the analysis plan before data analysis: the additional adjustment for cigarette dependence using the Heaviness of Smoking Index (HSI) [42]. Adjusted regression models are therefore reported with and without adjustment for HSI.

Data were analysed in R Studio. Data were weighted for prevalence statistics using a rim (marginal) weighting technique. This involves an iterative sequence of weighting adjustments whereby separate nationally representative target profiles are set (for gender, working status, children in the household, age, social grade and region). This process is then repeated until all variables match the specified targets. Unweighted data were used for the analyses below.

To address aim one, logistic regression was used to assess differences in e‐cigarette use and NRT use between the ethnic groups of interest, adjusting for age, gender, cigarette dependency (HSI) and socio‐economic status. The most prevalent category, White ethnicity, was used as the reference category.

Bayes factors (BFs) were derived for non‐significant findings using an online calculator to disentangle whether there was evidence for the null hypothesis of no effect (BF <⅓) or the data were insensitive (BF between ⅓ and 3). A normal distribution was assumed with an expected effect size of 0.6 (i.e. those of non‐White ethnicity have a 40% lower odds of using e‐cigarettes than those of White ethnicity) based on previous research [28].

To address aim two, aggregated prevalence of e‐cigarette use and NRT use was derived for each ethnic group as a function of year (although monthly data are available, sample sizes were too small for some ethnic minority groups to examine monthly trends). A trend analysis was then conducted to assess whether changes over time were different among the different ethnic groups. First, time was regressed onto prevalence of e‐cigarette use and NRT use in a simple linear regression model. Independent sample *t* tests then compared the slopes for each ethnic group relative to those of White ethnicity only. Because this was an exploratory analysis, α was set to 0.10 [43].

Where there was evidence of a significant difference in linear trends several additional models were assessed: (i) polynomial regression with terms up to an order of three (i.e. quadratic trend and cubic trend model); (ii) power regression (log–log model or power trend model); (iii) exponential regression (log‐level model or exponential trend model); and (iv) logarithmic regression (level‐log model or logarithmic trend model). We excluded other functions *a priori* (e.g. quartic and quantic polynomial regressions,) because we did not believe they reflected plausible underlying trends in prevalence indicators and could lead to overfitting.

To identify the best overall models, all the resulting regression models were compared using the Akaike information criterion (AIC) as the primary measure of fit, and the Bayesian information criterion (BIC) as a secondary measure of fit. A prerequisite in using the AIC and BIC to compare models is that the dependent variable is on the same scale; therefore, to ensure equivalence for the exponential trend and power trend models a correction was applied to the AIC and BIC. This involves adding the Jacobian of the log transformation (i.e. $2\sum_{i}\log\left(y_{i}\right)$ where y is the outcome variable of interest). The criteria for selecting the best fitting model was either the model with the lowest AIC, or the simplest model within two units of the model with the lowest AIC score. Orthogonal polynomials were used for model selection because they are uncorrelated, but raw polynomials are reported for the final models.

## RESULTS

### Participants

Between April 2013 and September 2019 data were collected on 132 597 adults of age 16+. Of these, 18.2% (95%CI 16.2 to 20.2; *n* = 24 114) were current smokers. Smoking prevalence differed as a function of ethnic group. It was highest among those of mixed/multiple ethnicity (22.8%, *n* = 416), followed by those of Arab/other ethnicity (20.0%, *n* = 324) and those of White ethnicity (19.0% *n* = 21 637). Prevalence was lowest among those of Black (11.2%, *n* = 498) and Asian ethnicity (11.1%, *n* = 1154).

### Association between ethnicity and use of e‐cigarettes and NRT for cutting down and temporary abstinence by current smokers

A total of 18% (95%CI 16.3–20.3, *n* = 4409) of current smokers reported using e‐cigarettes for cutting down and temporary abstinence and 9.20% (95%CI 7.2–11.1, *n* = 2213) were using NRT for cutting down and temporary abstinence. Table 1 shows the prevalence of e‐cigarette use and NRT use as a function of demographic characteristics. Tables 2 and 3 show the results of unadjusted and adjusted logistic regression analyses on this data.

**Table 1:** Prevalence of ANDS use for cutting down and temporary abstinence as a function of demographic characteristics.

	*Current e‐cigarette use for cutting down and temporary abstinence*		*Current NRT use for cutting down and temporary*	
	*n*	*%*	*n*	*%*
Ethnicity				
White	3895	18.3	1963	9.2
Mixed/multiple	76	17.2	51	11.5
Asian	190	14.7	121	9.4
Black	90	15.5	61	10.5
Arab/other	35	10.6	26	7.9
Female	2032	18.3	1099	9.9
Social grade				
AB	611	20.7	336	11.4
C1	1307	19.7	594	9.0
C2	971	17.6	484	8.8
D	784	16.7	425	9.0
E	625	14.8	390	9.2
Owns home	1745	18.8	872	9.4
Age				
16–24	802	17.6	265	5.8
25–34	907	19.1	407	8.6
35–44	759	19.8	409	10.6
45–54	759	18.9	438	10.9
55–65	641	18.5	384	11.1
65+	416	12.7	319	9.7
HSI				
0–2 Low addiction	2662	17.6	1335	8.8
3–4 Moderate addiction	1306	18.7	745	10.6
5–6 High addiction	150	19.5	85	11.1

Note: age and HSI were entered into the models as continuous variables but for descriptive purposes are presented as categorical above. ANDS = alternative nicotine delivery systems; NRT = nicotine replacement therapy.

**Table 2:** Results of the unadjusted regression analysis assessing the association between ethnicity and ANDS use for harm reduction.

	*Current e‐cigarette use for cutting down and temporary abstinence*				*Current NRT use for cutting down and temporary abstinence*			
	*OR*	*95% CI*	*P*	*BFs*	*OR*	*95% CI*	*P*	*BFs*
White	1				1			
Mixed/multiple	0.93	0.72–1.19	0.571	0.3	1.29	0.96–1.73	0.096	1.1
Asian	0.77	0.66–0.90	0.001	31.5	1.02	0.84–1.24	0.840	0.2
Black	0.82	0.65–1.03	0.085	0.9	1.15	0.88–1.51	0.298	0.4
Arab/other	0.53	0.37– 0.76	<0.001	67.3	0.85	0.57–1.27	0.415	0.5

BF = Bayes factors. ANDS = alternative nicotine delivery systems; NRT = nicotine replacement therapy.

**Table 3:** Results of the adjusted regression analysis assessing the association between ethnicity and ANDS use for cutting down and temporary abstinence.

	*Current e‐cigarette use for cutting down and temporary abstinence*						*Current NRT use for cutting down and temporary abstinence*							
	*Model 1*			*Model 2*			*Model 1*				*Model 2*		
	*AOR*	*95% CI*	*P*	*AOR*	*95% CI*	*P*	*AOR*	*95% CI*	*P*	*BFs*	*AOR*	*95% CI*	*P*	*BFs*
Ethnicity														
White	1			1			1				1			
Mixed/multiple	0.88	0.68–1.13	0.313	0.93	0.72–1.20	0.589	1.39	1.03–1.87	0.033	2.5	1.42	1.04–1.94	0.027	2.7
Asian	0.74	0.63–0.87	<0.001	0.79	0.66–0.93	0.005	1.18	0.97–1.44	0.100	0.7	1.21	0.98–1.49	0.069	0.9
Black	0.80	0.63–1.01	0.057	0.87	0.68–1.11	0.255	1.17	0.89–1.55	0.264	0.5	1.21	0.90–1.62	0.200	0.6
Arab/other	0.51	0.36–0.73	<0.001	0.58	0.40– 0.83	0.003	0.93	0.62–1.39	0.725	0.4	1.04	0.69–1.56	0.851	0.4
Female	1.05	0.98–1.12	0.186	1.05	0.98–1.13	0.163	1.16	1.06–1.27	0.001		1.14	1.04–1.25	0.005	
Social grade														
AB	1			1			1				1			
C1	0.92	0.82–1.03	0.140	0.90	0.80–1.01	0.063	0.77	0.67–0.89	<0.001		0.75	0.64–0.86	<0.001	
C2	0.81	0.73–0.91	0.001	0.76	0.67–0.85	<0.001	0.75	0.64–0.87	<0.001		0.69	0.59–0.81	<0.001	
D	0.77	0.68 – 0.97	<0.001	0.69	0.61 –0.79	<0.001	0.77	0.66 –0.90	0.001		0.71	0.60–0.83	<0.001	
E	0.68	0.60 –0.78	<0.001	0.61	0.54 –0.70	<0.001	0.74	0.63 –0.88	<0.001		0.67	0.57 –0.80	<0.001	
Owns home	1.10	1.02 –1.18	0.017	1.11	1.02 –1.19	0.011	0.88	0.80 –0.98	0.017		0.90	0.82 –1.00	0.055	
Age	0.99	0.99 –0.99	<0.001	0.99	0.99 – 0.99	<0.001	1.01	1.01 – 1.01	<0.001		1.01	1.01 – 1.01	<0.001	
HSI				1.08	1.05 –1.10	<0.001					1.10	1.06 –1.13	<0.001	

BF = Bayes factors. ANDS = alternative nicotine delivery systems; NRT = nicotine replacement therapy.

Odds of current e‐cigarette use for cutting down and temporary abstinence was significantly lower at 21% and 42% compared with White ethnicity among those of Asian ethnicity and Arab/other ethnicity, respectively. Those of mixed/multiple ethnicity had 42% higher odds of current NRT use for cutting down and temporary abstinence than those of White ethnicity. BFs indicated that the data were largely insensitive to detect an OR of 0.6 or 1.7 for non‐significant findings with only anecdotal evidence for the null hypothesis (see Tables 2 & 3).

Trends over time in use of e‐cigarettes and NRT for cutting down and temporary abstinence by current smokers as a function of ethnicity found that for smokers of White ethnicity, current e‐cigarette and NRT use followed cubic trends. The former was characterised by an ‘inverse S’ shaped curve with an increase in e‐cigarette prevalence that slowed over time up until 2015, which was then followed by a steady decline for the rest of the series. The latter was characterised by an ‘S’ shaped curve with a small decline in NRT for cutting down and temporary abstinence up until approximately 2015, followed by a period of stability and then a small decline between 2018 and 2019. NRT also followed a cubic trend for those of mixed/multiple ethnicity, characterised by a similarly patterned ‘S’ shaped curve as for White smokers. For all other ethnic groups, trends were non‐significant, suggesting little change over time, although the small sample size in some subgroups may also have meant that the data were insensitive to detect any effect (see Tables 4 and 5; Supporting information Figs. S1 & S2).

**Table 4:** Indices of fit for the trend models.

	*White ethnicity*		*Mixed/multiple ethnicity*		*Asian ethnicity*		*Black ethnicity*		*Arab/other ethnicity*	
	*AIC*	*BIC*	*AIC*	*BIC*	*AIC*	*BIC*	*AIC*	*BIC*	*AIC*	*BIC*
Current e‐cigarette use										
Linear model	23.9	23.7	51.5	51.4	38.4	38.2	44.1	43.9	47.3	47.2
Quadratic model	18.3	18	53.5	53.3	32.2	32.0	42.7	42.5	45.1	44.9
Cubic model	8.0	7.7	53.9	53.6	31.7	31.5	43.9	43.7	46.9	46.6
Logarithmic model	24.2	24.1	51.5	51.4	40.0	39.9	43.1	43.0	47.5	47.3
Exponential model	23.7	23.6	53.4	53.3	38.5	38.4	46.2	46.1	46.9	46.7
Power model	24.1	23.9	53.5	53.3	40.0	39.9	45.6	45.4	47.0	46.8
Current NRT use										
Linear model	31.6	31.4	43.9	43.7	31.5	31.4	45.5	45.3	45.4	45.2
Quadratic model	29.4	29.2	45.9	45.6	32.6	32.4	42.0	41.8	45.9	45.6
Cubic model	20.5	20.2	33.9	33.6	34.2	33.9	43.6	43.3	45.6	45.3
Logarithmic model	29.5	29.3	43.5	43.3	31.0	30.8	43.4	43.2	45.1	45.0
Exponential model	30.5	30.3	47.7	47.6	34.5	34.4	42.7	42.5	44.1	44.0
Power model	28.7	28.5	48.6	48.5	34.3	34.1	41.1	41.0	44.0	43.8

AIC = Akaike information criterion; BIC = Bayesian information criterion; NRT = nicotine replacement therapy.

**Table 5:** Trend analysis results.

	*White ethnicity*			*Mixed/multiple ethnicity*			*Asian ethnicity*			*Black ethnicity*			*Arab/other ethnicity*		
	*B*	*95% CI*	*P*	*B*	*95% CI*	*P*	*B*	*95% CI*	*P*	*B*	*95% CI*	*P*	*B*	*95% CI*	*P*
Current e‐cigarette use															
Time	4.91	1.92 to 7.89	0.014	1.38	−2.21 to 4.97	0.369	0.96	−0.44 to 2.37	0.138	−1.07	−3.18 to 1.05	0.251	6.90	−4.33 to 18.13	0.163
Time^2	−1.19	−2.03 to −0.350	0.020										−0.90	−2.27 to 0.47	0.142
Time^3	0.08	0.01 to 0.15	0.032												
Current NRT use															
Log (Time)										−0.51	−1.03 to 0.01	0.054			
Time	10.30	−17.58 to −3.02	0.020	−26.94	−45.94 to −7.94	0.020	−0.63	−1.49 to 0.23	0.119				0.47	−1.85 to 2.78	0.626
Time^2	2.43	0.38 to 4.48	0.032	7.27	1.92 to 12.61	0.023									
Time^3	−0.18	−0.35 to −0.01	0.044	−0.60	−1.05 to −0.16	0.022									

NRT = nicotine replacement therapy.

## DISCUSSION

### Summary of findings

Odds of self‐reported current e‐cigarette use for cutting down and temporary abstinence among smokers were 21% lower among smokers of Asian ethnicity and 42% lower among those of Arab/other ethnicity compared with those of White ethnicity. Smokers of mixed/multiple ethnicity had 42% higher odds of current NRT use for cutting down and temporary abstinence. Trend analysis showed differences in the shapes of trajectories in prevalence since 2013. Self‐reported e‐cigarette and NRT use by smokers for cutting down and temporary abstinence followed cubic trends for those of White ethnicity; the first being characterised by an ‘inverse S’ shaped curve and the latter an ‘S’ shaped curve. NRT use by smokers for cutting down and temporary abstinence over time also followed an ‘S’ shaped curve for those of mixed/multiple ethnicity. For all other ethnic groups non‐significant trends were found.

### Comparison to previous studies

These findings are consistent with previous studies, which have shown that smokers from some ethnic minority groups are less likely than those of White ethnicity to use e‐cigarettes and NRT [26, 27, 28, 29, 30, 31, 32, 33]. This could potentially be because those from ethnic minority groups hold more negative attitudes towards treatment and concerns about medication adverse effects [44, 45].

Those of mixed/multiple ethnicity had the highest smoking prevalence rate. The mixed/multiple category is the fastest growing ethnic group in the United Kingdom and there have been several calls to consider their potential uniqueness in terms of health behaviours when making policy decisions [46]. An interesting finding was that use of NRT was greatest among those of mixed/multiple ethnicity. This suggests a possibly higher level of acceptance of evidence‐based medication in this group. It could also be that other ethnic groups prefer other evidence‐based options that were not assessed here (e.g. behavioural support, varenicline and bupropion).

In contrast, those of Arab/other ethnicities were substantially less likely to opt for the use of e‐cigarettes, despite having the second highest smoking rates. Research is needed to explore this further to help reduce social inequalities in health.

The difference in the trajectories of e‐cigarette prevalence over time as a function of ethnicity suggests that there may be a need to consider stratification in future studies, particularly if evidence is being used to inform population‐level policy and research priorities. Whereas for those of White ethnicity there is a need to identify the factors that may be contributing to a decline in ANDS use in recent years, the most obvious one being misperceptions because of the media about the harms of e‐cigarettes, a stronger research priority for other ethnic minority groups may be to focus on understanding the reasons for the low prevalence of use over time [47, 48].

### Implications

Ethnic minority groups in England appear to be at a higher risk for a number of smoking‐related diseases [49]. Guidance by the NICE has noted that reducing smoking prevalence among some ethnic minority groups could reduce health inequalities more so than any other measure [50]. Previous studies have shown the efficacy of ANDS for cutting down and temporary abstinence [4, 9, 21], mainly for NRT, therefore the finding that they are currently underused among ethnic minority groups compared with White smokers indicates an area of possible intervention. Of course, continued monitoring of the effectiveness of ANDs is needed particularly as evidence suggests that the direction of association between use for cutting down and temporary abstinence and cessation may depend on the device type and also frequency of use [51, 52].

### Strengths and limitations

This study used a large representative survey of the adult population in England to assess the association between e‐cigarette and NRT use for cutting down and temporary abstinence and ethnicity. First, as with all cross‐sectional observational surveys, caution should be taken when interpreting these findings because there is a possibility that unmeasured covariates could have led to artificial associations. Second, although this paper assessed two measures of socio‐economic status, social grade and housing tenure, which are the best predictors of smoking status [53], there are several limitations with the use of these [54, 55]. Third, although the sample was designed to be representative, there is a risk of bias in terms of the characteristics of those who agree to participate. There is also a risk that respondents may fail to report their smoking status. Fourth, we had to collapse categories across some ethnicities because of sample size. Therefore, there may be important within‐category differences that were not identified in the current study. For example, smoking behaviour in the mixed/multiple ethnic group according to the Continuum of Biracial Identity (COBI) model [56] is likely to mirror where an individual sees themselves on an ethnic identify continuum. If they identify more with those of White ethnicity, for example, their behaviour will be more closely aligned with that population. Fifth, this study did not assess the effectiveness of attempts at cutting down and temporary abstinence by smokers. Although studies have shown previously that NRT use and e‐cigarette use while concurrently smoking is associated with greater success of quit attempts, this may not apply across ethnic groups [8, 9, 57]. It will be important to investigate this further as a larger sample size is accumulated. Sixth, the sample size for some categories also means that caution should be taken when interpreting the results of the trend analysis. A non‐significant trend may be because of data insensitivity or evidence for the null of no changes over time [58, 59]. Seventh, these findings may not be applicable to other countries with different compositions of ethnic groups or where tobacco control climates are different to England. Finally, this paper did not consider differences in the use of e‐cigarettes and NRT as an aid to smoking cessation. This has been the focus of previous studies but still warrants investigation.

### CONCLUSION

In England, e‐cigarette use for cutting down and temporary abstinence is less common among smokers of Asian than those of White ethnicity. Smokers of mixed/multiple ethnicity are the most likely to be using NRT. Significant cubic trends for the prevalence of e‐cigarette use and NRT use over time were found for those of White ethnicity (‘inverse S’ shaped curve for e‐cigarettes and ‘S’ shaped curve for NRT) and for NRT use among those of mixed/multiple ethnicity. Prevalence of use was more stable in other ethnic groups.

## Ethical approval

Ethical approval for the Smoking Toolkit Study was originally granted by the UCL Ethics Committee (ID 0498/001). Explicit verbal agreement and willingness to answer questions voluntarily is recorded electrically by Ipsos Mori, the company administering the survey. This is standard protocol and was agreed by the UCL ethics committee. Participants are also given a printed information sheet.

## Declaration of interests

L.S. undertake consultancy and research for and receive travel funds and hospitality from manufacturers of smoking cessation medications. E.B., J.B., H.T.B. and L.S. have received unrestricted research funding from Pfizer.

## Funding

The Smoking Toolkit Study is currently primarily funded by Cancer Research UK (C1417/A14135; C36048/A11654; C44576/A19501) and has previously also been funded by Pfizer, GSK and the Department of Health. No funders had any involvement in the design of the study, the analysis or interpretation of the data, the writing of the report or the decision to submit the paper for publication. E.B., S.J., and J.B. are funded by Cancer Research UK (C1417/A14135). H.T.B.’s studentship is funded by Public Health England. The views expressed are those of the authors and not necessarily those of Cancer Research UK or other funders. No funders had any involvement in the design of the study, the analysis or interpretation of the data, the writing of the report or the decision to submit the paper for publication.

## Author contributions

**Emma Beard:** Conceptualization; formal analysis; methodology. **Jamie Brown:** Conceptualization; methodology. **Sarah Jackson:** Conceptualization; methodology. **Harry Tattan‐Birch:** Conceptualization; methodology. **Lion Shahab:** Conceptualization; methodology.

## Supporting information

**Figure S1** Fitted models for the trend analysis of current e‐cigarette use for harm reduction among those of (a) White ethnicity, (b) mixed/multiple ethnicity, (c) Asian ethnicity, (d) Black ethnicity and (e) Arab/other ethnicity.
**Figure S2** Fitted models for the trend analysis of current NRT use for harm reduction among those of (a) White ethnicity, (b) mixed/multiple ethnicity, (c) Asian ethnicity, (d) Black ethnicity and (e) Arab/other ethnicity.
