# Supplementary material for: Differences between ethnic groups in self‐reported use of e‐cigarettes and nicotine replacement therapy for cutting down and temporary abstinence: a cross‐sectional population‐level survey in England
Source: Addiction. 2021 Mar 18;116(9):2476–85. doi: 10.1111/add.15431 (PMC8438700; doi:10.1111/add.15431)
Supplement: Supplementary file 1 — Figure S1 Fitted models for the trend analysis of current e‐cigarette use for harm reduction among those of (a) White ethnicity, (b) mixed/multiple ethnicity, (c) Asian ethnicity, (d) Black ethnicity and (e) Arab/other ethnicity. Figure S2 Fitted models for the trend analysis of current NRT use for harm reduction among those of (a) White ethnicity, (b) mixed/multiple ethnicity, (c) Asian ethnicity, (d) Black ethnicity and (e) Arab/other ethnicity. [file ADD-116-2476-s001.docx]

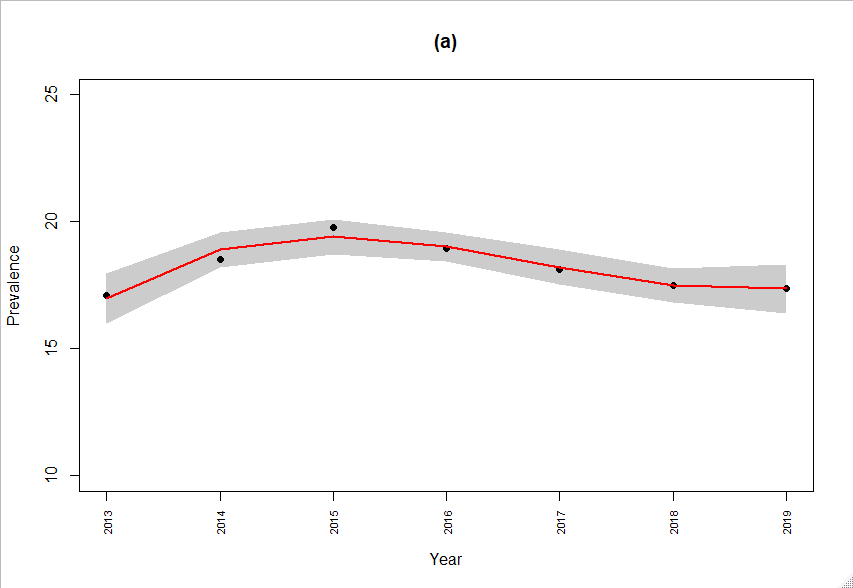

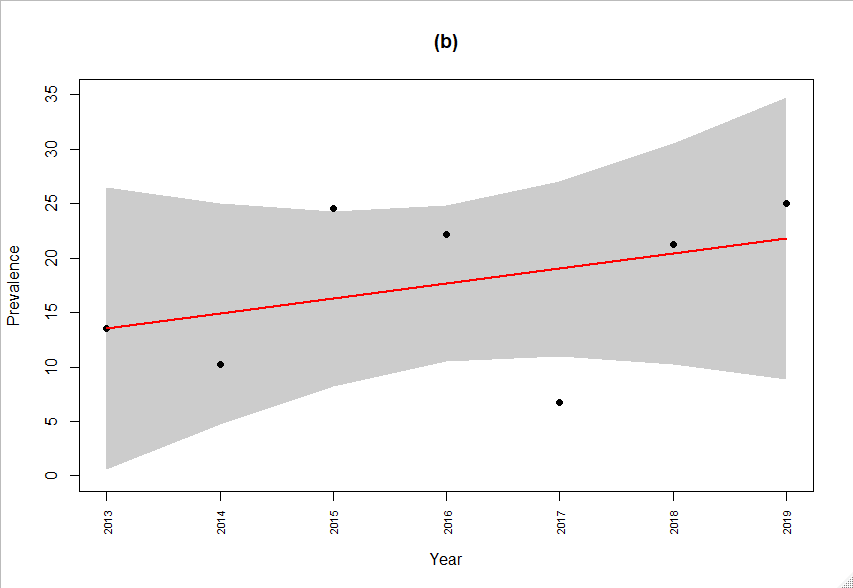

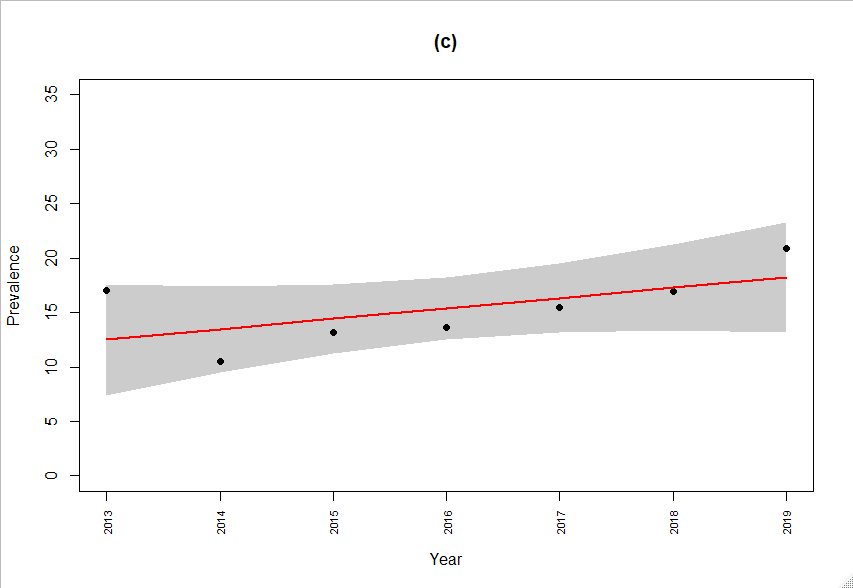

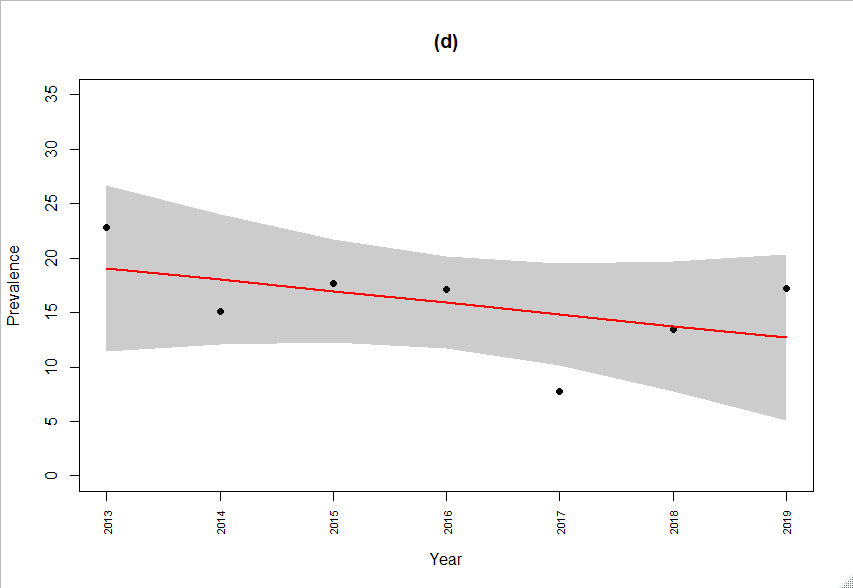

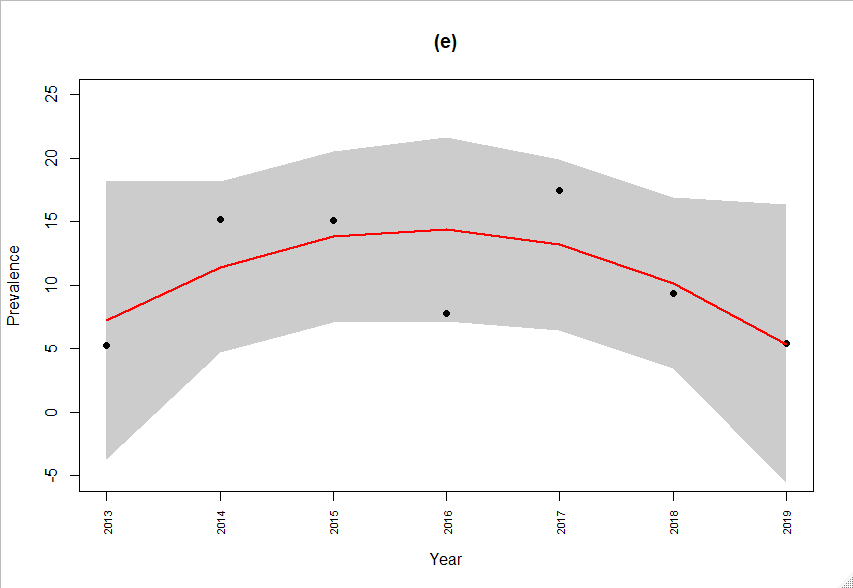


**Supplementary Figure 1:**  Fitted models for the trend analysis of current e-cigarette use for harm reduction among those of (a) White ethnicity, (b) mixed/multiple ethnicity, (c) Asian ethnicity, (d) Black ethnicity and e) Arab/other ethnicity


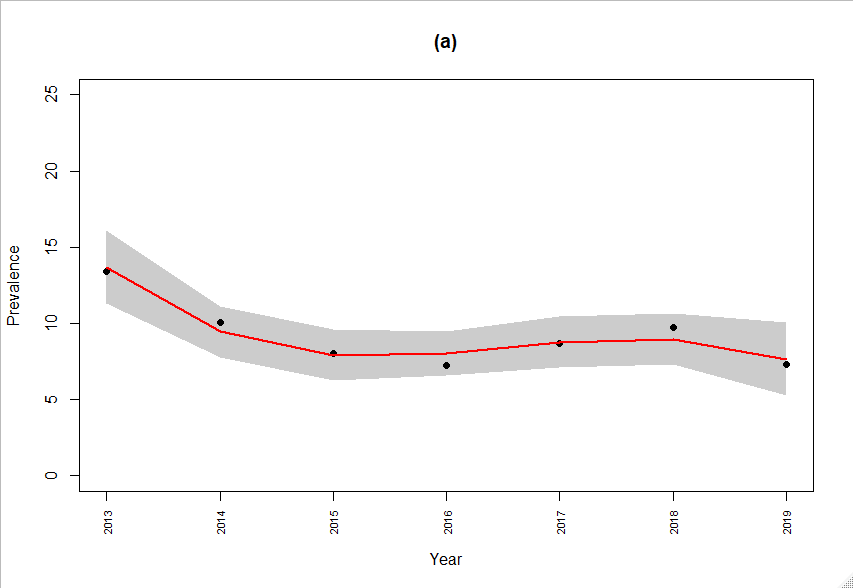

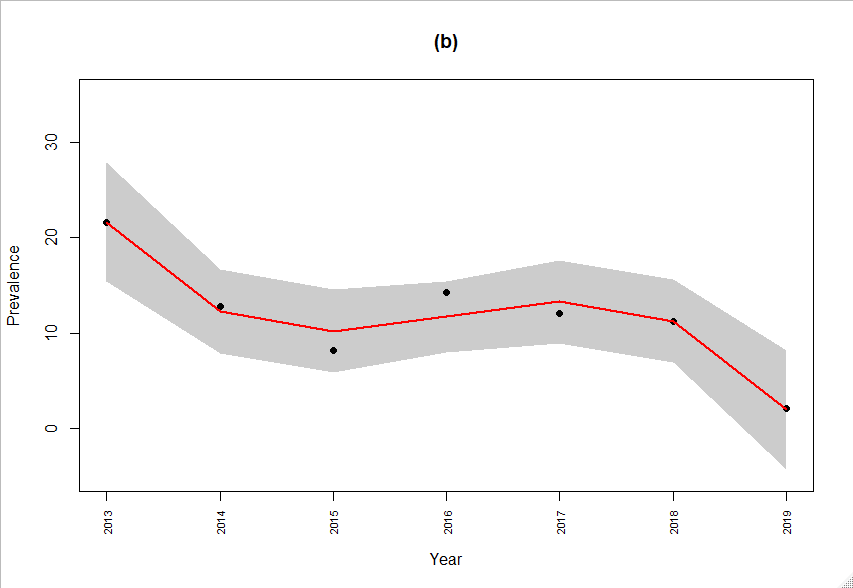

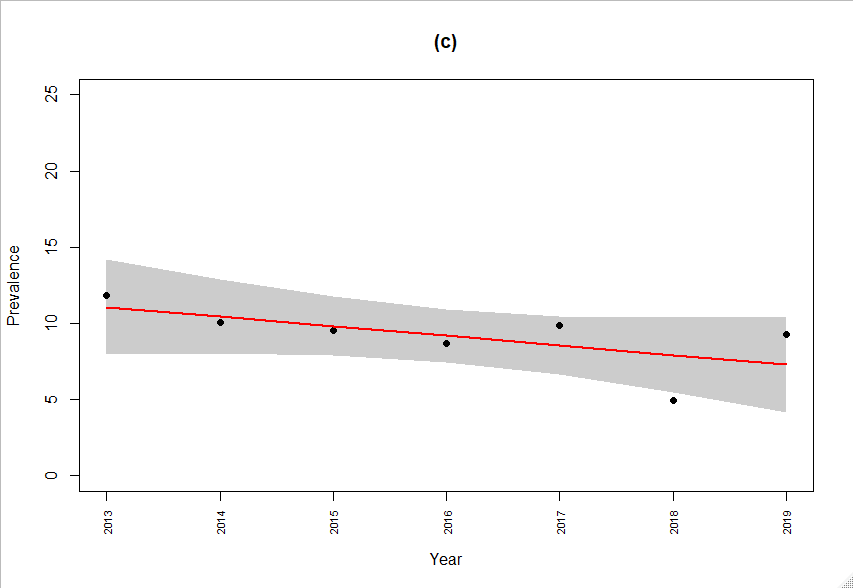

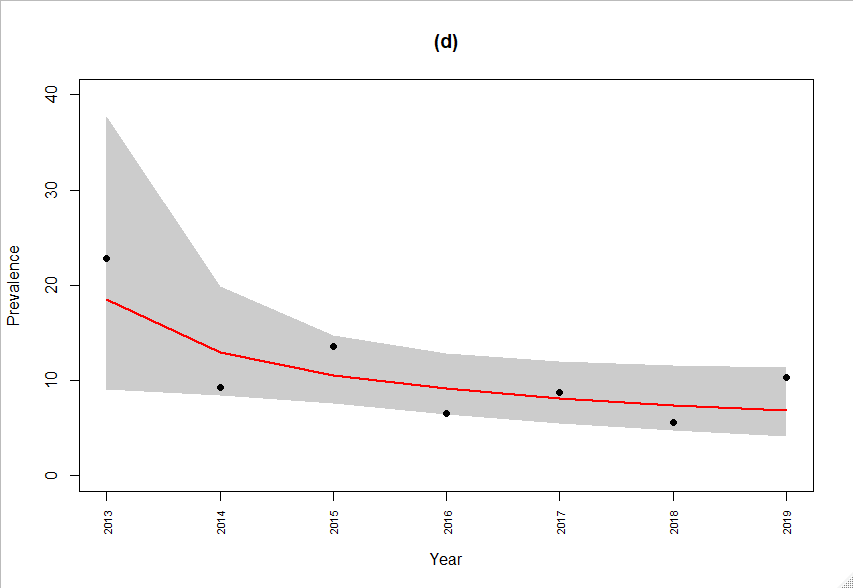

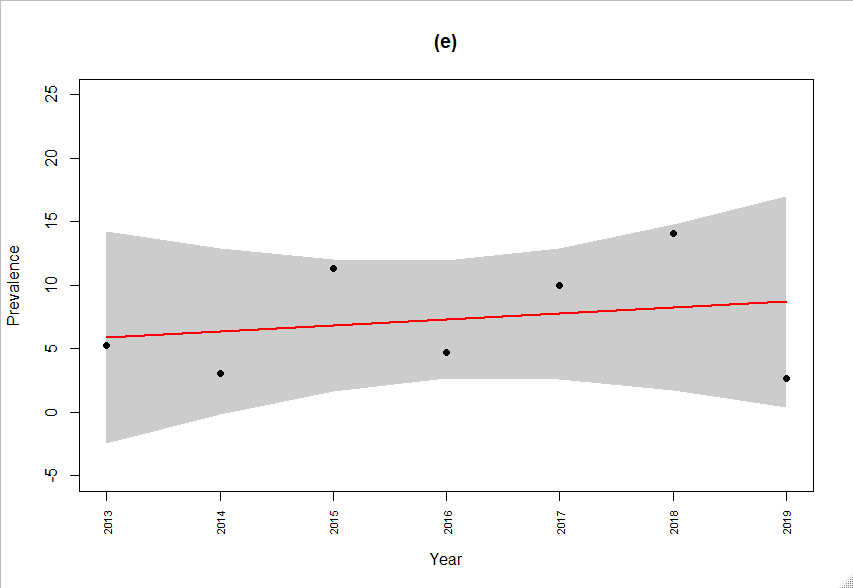


**Supplementary Figure 2:**  Fitted models for the trend analysis of current NRT use for harm reduction among those of (a) White ethnicity, (b) mixed/multiple ethnicity, (c) Asian ethnicity, (d) Black ethnicity and e) Arab/other ethnicity
